# Serum hsa_circ_0087776 as a new oncologic marker for the joint diagnosis of multiple myeloma

**DOI:** 10.1080/21655979.2021.2005875

**Published:** 2021-12-14

**Authors:** Xingxing Gong, Xu Lu, Jing Cao, Huan Liu, Hongmei Chen, Fang Bao, Xiuying Shi, Hui Cong

**Affiliations:** a Department of Laboratory Medicine, Affiliated Hospital of Nantong University, Nantong P.R. China; b Neurosurgery Department, Linqing People’s Hospital, Linqing, P.R. China; c Vip Ward, Affiliated Hospital of Nantong University, Nantong P.R. China; d Department of Blood Transfusion, Affiliated Hospital of Nantong University, Nantong P.R. China

**Keywords:** Multiple myeloma, hsa_circ_0087776, biomarker, serum, diagnosis, quantitative real-time PCR

## Abstract

Multiple myeloma (MM) is a hematologic malignancy caused by abnormal proliferation of bone marrow plasma cells, which lacks diagnostic markers and has a general prognosis. At present, the understanding of its pathogenesis provides the basis for the combined diagnosis and new targeted therapy of the disease. In this study, quantitative real-time PCR was used to detect 136 MM patients and 74 healthy controls, and the clinical application value of hsa_circ_0087776 as a new tumor marker and combined diagnosis was evaluated. The results showed that the expression of hsa_circ_0087776 was significantly lower in serum of MM patients (*P*-value < 0.0001), and the expression was consistent in MM cells. In the analysis of clinicopathological parameters, it was found that there were significant statistical differences with MM stage and renal injury. In addition, it significantly increased the sensitivity with ALB, β₂-MG joint diagnosis, to provide a basis for diagnosis, improve the prognosis of the disease, improve the survival of patients and quality of life. These studies suggest that hsa_circ_0087776 can be used as a new oncology marker for the combined diagnosis of MM.

**Impact statement:** Various evidences have shown that the role of circRNA in the occurrence and development of diseases is potentially unknown and untapped. Therefore, it has a broad prospect to find circRNA specifically expressed in MM patients for combined diagnosis and targeted therapy of MM. However, MM lacks such specific tumor markers. Therefore, the discovery of new specific tumor markers for combined diagnosis is an important milestone in the development of medical history. In the research, we founded hsa_circ_0087776 can be used as a new oncology marker for combined diagnosis of MM.

## Introduction

Multiple myeloma (MM) is a terminally differentiated plasma cell tumor that produces large amounts of immunoglobulin, accounting for 1% of all cancers and 10% of hematological tumors [1–3]. Most patients present with a wide range of systemic diseases, accompanied by important skeletal and renal involvement, and the mortality of MM among inpatients is third only to lung cancer and liver cancer [4,5]. In the body’s immune monitoring, Natural killer cells (NK) as a kind of natural immune effect factor, can directly identify and kill MM cells [6], so as to protect the body, when the immune system protection less than tumor cell damage is there will be a degree of different clinical manifestations, and because of the difference of MM patients with genome, varied clinical manifestations [7]. With the advent of new targeted drugs and the combined application of drugs, the prognosis of MM patients has been greatly improved, but studies have shown that up to 22% of patients receiving targeted therapy have developed renal failure [8]. Renal function impairment is one of the inevitable side effects during the use of chemotherapy drugs. Timely detection of MM, clinical intervention as soon as possible can greatly improve the prognosis of MM patients and effectively reduce the mortality of patients. Now β₂-MG, ALB can be used as MM auxiliary diagnostic criteria, but there is still a lack of effective biological markers. Therefore, it is urgent to find new tumor markers.

Circular RNAs (CircRNAs) are a class of endogenous non-coding RNAs (ncRNAs) with a closed-loop structure formed by antisplicing of precursor mRNA. Because they lack a 5ʹ – end cap and a 3ʹ – end poly(A) tail, they are resistant to exonuclase digestion and have stronger stability than mRNA, microRNA (miRNA) and long non-coding RNAs (lncRNAs) [9–11], it also has the characteristics of abundant species, widespread existence, conserved sequence and stable expression in cells and tissues [12]. Studies have found that it has a clear correlation with the proliferation, metastasis and apoptosis of tumor cells, can regulate the occurrence and development of tumors, and is used in the prediction, diagnosis, treatment and prognosis of a variety of diseases [13]. Various evidences have shown that the role of circRNA in the occurrence and development of diseases is potentially unknown and untapped [14]. Therefore, it has a broad prospect to find circRNA specifically expressed in MM patients for combined diagnosis and targeted therapy of MM.

Yu et al reported that circ-MyBL2, as a tumor suppressor gene, affects tumor progression by inhibiting MM cell viability, DNA synthesis and cell cycle progression, and serves as a novel tumor marker [15]. Subsequently, with the advent of circ_0000142, circ-cdyl, and hsa_circRNA_101237 [16–18], infinite possibilities were provided for the prospect of MM treatment. Alpha-fetoprotein (AFP) in hepatocellular carcinoma (HCC) cells can be used as indicators of disease diagnosis, recurrence and so on, which significantly changes the survival time of patients [19]. Carcinoembryonicantigen (CEA) has also made a significant contribution to the early diagnosis of tumors [20]. However, MM lacks such specific tumor markers. Therefore, the discovery of new specific tumor markers for combined diagnosis is an important milestone in the development of medical history. We found low expression of hsa _circ _0087776 in MM cells in the preliminary experiment, and we speculated that hsa _circ _0087776 may also show low expression in MM patients. Detection alone or in combination with other markers can improve diagnostic efficacy and evaluate efficacy.

## Materials and methods

### Study subjects

Serum samples were collected from 136 patients with multiple myeloma (excluding patients with other cancers and primary renal disease), Sixteen of them were newly diagnosed patients, In addition 74 healthy controls (age and region matched) who were admitted to the Affiliated Hospital of Nantong University from September 2019 to February 2021. Serum samples were collected according to the blood collection procedure of the Affiliated Hospital of Nantong University. The blood sample was centrifuged at 3000 g for 10 min. Upper serum of 900 µl was extracted and divided equally 1.5 mL RNase-free EP tubes, and stored at −80°C for use. The Ethics Committee of Affiliated Hospital of Nantong University permitted this study.

#### Real-time quantitative PCR (qRT-PCR)

Total RNA was extracted from 300 μl serum by using a serum extract Kit (Life Technologies, USA) according to the manufacturer’s protocol. The extracted total RNA was reverse-transcribed into single-stranded cDNA using a reverse transcription kit (Thermo Fisher Science, US) according to the manufacturer’s instructions [21]. The expression of hsa_circ_0087776 in serum samples was detected by qRT-PCR. The hsa_circ_0087776 and 18S primers were designed by Ribaud Corporation (Guangzhou, China). The hsa_circ_0087776 primer sequence: 5´-AATGAATGCTTTCTCAGAAGGG – 3´ (forward);5´ – AGACTCCCTCACATTTAATTCTCCT-3´ (reverse). The 18S rRNA primer sequences are: 5´-GTAACCCGTTGAACCCAT-3´(forward); 5´-CCATCCAATCGGTAGTAGCG-3´ (reverse). We used 18s as an internal reference to conduct standard quantification of expression in all samples, and the above experiment was repeated three times.With 2 ^– ΔΔCT^ method to calculate the relative expression of the hsa_circ_0087776.

#### Agarose gel electrophoresis

1.5 g agarose was added to 50 mL 1 × Tris Acidate-EDTA (TAE) solution to prepare the gel, which was microwaved until dissolved, Add 2 μl ethidium bromide after cooling. Placed the solidified gel in the electrophoresis tank, mix 5 μl of PCR product and 1 μl of buffer solution respectively, and add into the sample well. Electrophoresis parameter setting: voltage (V) – 110; Ampere (A) – 210; The time was about 40 min, and the results were observed with a gel imaging system.

#### Cell culture

HS-5 (human stromal cell) and MM myeloma cells H929, U266 and RPMI-8226 with very low differentiation degree were selected (purchased from Cell Bank of Chinese Academy of Sciences, Shanghai, China). 10% fetal bovine serum (GIBCO fetal bovine serum from Big Island, New York) was cultured at 37°C in a 5% carbon dioxide incubator [22]. After the cell density reached about 80%, the cells were centrifuged, 1 mL Trizol was added to the lower cell precipitates, beaten and mixed, and stored in a refrigerator at −80°C for later use [23].

#### RNase R treatment

According to the above steps, RNA was extracted from cells and divided into 2 μg equal amount into two 1.5 mL RNase-free EP tubes. One was added to 8 U RNase R for 20 min at 37°C and the other was not processed. Single-stranded cDNA was obtained through reverse transcription, and the expression level of relevant hsa_circ_0087776 was detected by qRT-PCR [24].

#### Statistical analysis

Statistical analysis of clinicopathological parameters was performed using SPSS 24.0 (IBM 226 Corporation, Armonk, USA), and graphics were produced by GraphPad Prism 8.0 (GraphPad Software Inc., USA). One-way analysis of variance and independent sample *t* test were used for statistical analysis. The clinical diagnostic efficacy of hsa_circ_0087776, ALB, β₂-MG was evaluated by AUC area under ROC curve. *P* < 0.05 indicated a statistical difference.

## Results

Here, we intended to explore the application of hsa_circ_0087776 in the diagnosis and efficacy evaluation of MM patients. A series of in vitro experiments, we found that hsa_circ_0087776 has good stability and low expression in MM, and its expression level is related to treatment, which has the potential to be used as a biomarker for the diagnosis and efficacy observation of MM.

### Methodological evaluation of serum hsa_circ_0087776

**Figure 1.**
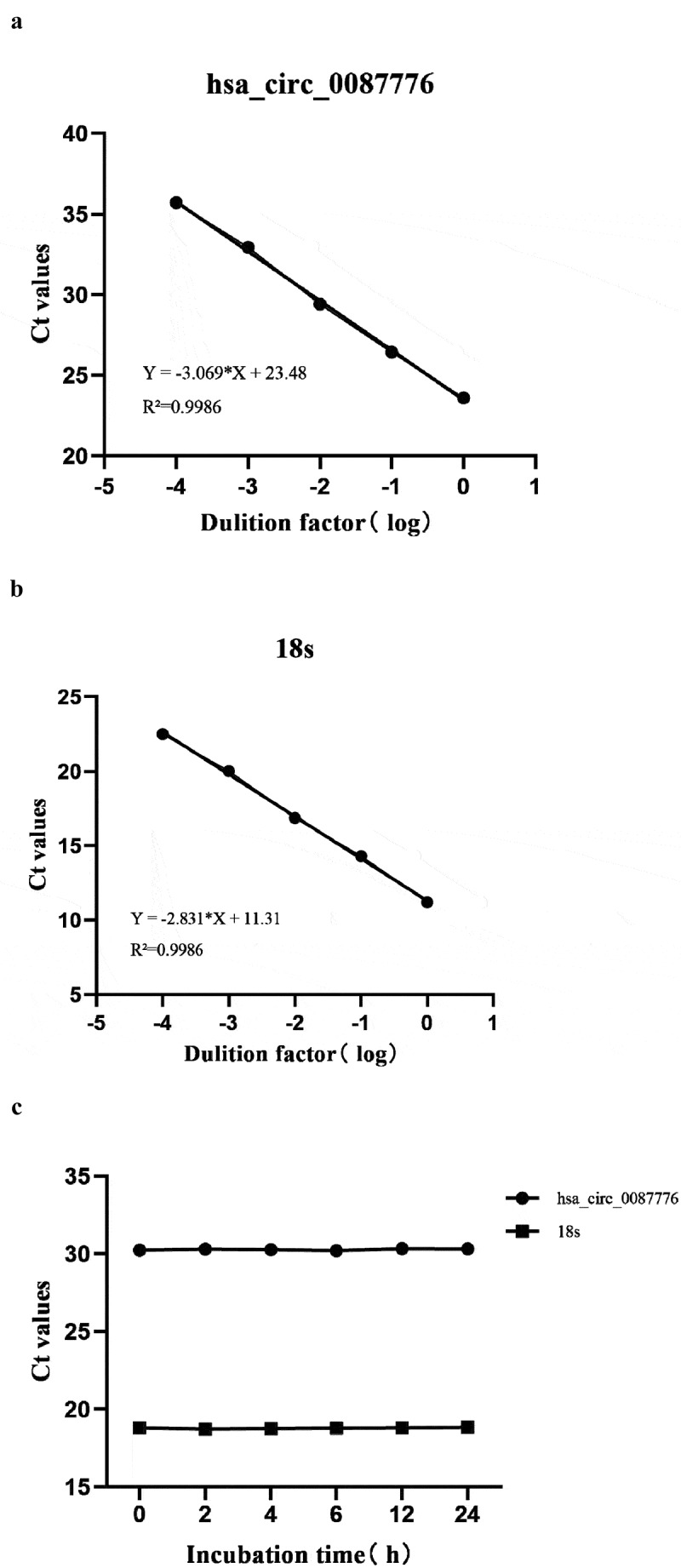
Methodological evaluation of hsa _circ _0087776. (a) The hsa_circ_0087776 standard curve. (b) The 18s standard curve. (c, d) Stability of hsa_circ_0087776 and 18s in room temperature incubation and repeated freeze-thaw

Bone marrow cell biopsy is the gold standard for the diagnosis of MM, but it is too traumatic to be widely used in clinical practice. The emergence of tumor cytological markers is an important milestone in the history of medicine.In order to verify the reliability of qRT-PCR methodology, cDNA in serum samples from MM patients was diluted by 10 times ratio in this study to compare whether the changes in CT values were linear. The results show that R^2^ = 0.9986 in hsa_circ_0087776, and the standard curve is Y = −3.069*X + 23.48, shown in Figure 1(a). R^2^ = 0.9986 in internal reference 18s. The standard curve is Y = −2.831*X + 11.31, R^2^ = 3.9986, shown in Figure 1(b). Both have good linear correlation and meet the experimental requirements.

In addition, 20 serum samples (including MM patients and healthy negative controls) were randomly selected, mixed and divided into 20 equal portions.Total RNA was extracted from 10 samples from the same batch. The in-batch coefficient of variation (CV) was calculated from the CT value.The remaining 10 samples were extracted at an average of one sample per day, and the corresponding inter-batch CV values were calculated. The test confirmed that the CV values within and between batches were less than 2%, show in Table 1, which met the requirements of experimental stability and reproducibility.

**Table 1.** The intra-assay and interassay repeatability difference of hsa_circ_0087776

	hsa _circ _0087776	18s
Intra-assay		
Mean ± SD	29.797 ± 0.358	19.650 ± 0.278
CV, %	1.07%	0.83%
Inter-assay		
Mean ± SD	30.209 ± 0.5576	17.823 ± 0.353
CV, %	1.67%	1.06%

Note: The intra-assay and inter-assay of hsa_circ_0087776 and 18 s were lower than 2%. CV: Coefficient of Variance.

**Figure 2.**
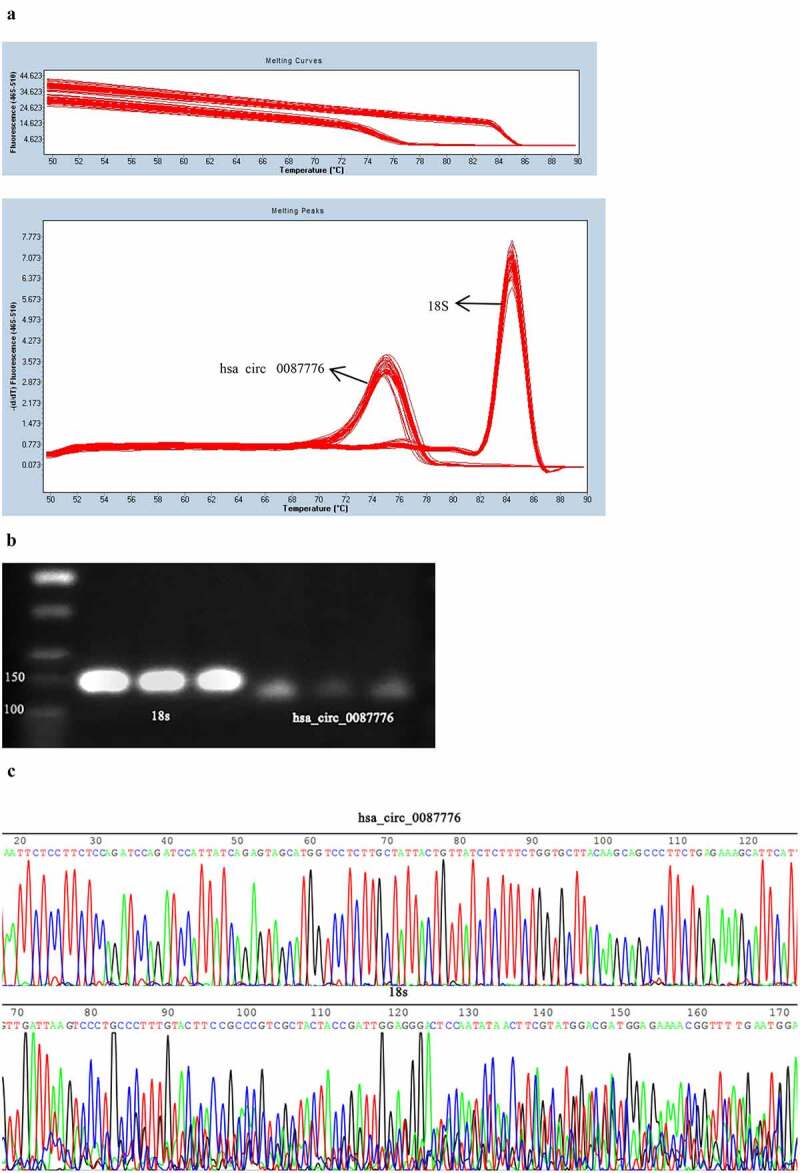
Methodological evaluation of hsa _circ _0087776. (a) Single peak specificity of hsa _circ _0087776 and 18s dissolution and amplification curves. (b) The specificity of hsa _circ _0087776 and 18s was verified by agarose gel electrophoresis. (c) Sequencing results of hsa _circ _0087776 and 18s

To verify the influence of experimental environment on molecular expression in serum, the serum was incubated at room temperature for 0, 2, 4, 6, 12, and 24 h. Repeated freeze-thaw 0, 3, 5, 10 times, and the experiment was repeated for three times. According to the comparison of CT values, the circRNA expression in the serum was stable, shown in Figure 1(c) and (D), and there was no statistical difference. 18S and hsa_circ_0087776 showed unimodal expression and good specificity, shown in Figure 2(a). Agarose gel electrophoresis results showed that 18S presented a single 151 bp band and hsa_circ_0087776 presented a single 126bp band, shown in Figure 2(b). Its correctness accords with the requirement of this experiment. Figure 2(c) shows that sanger sequencing results showed that the sequence homology of hsa_circ_0087776, 18S and NCBI was 100%. In conclusion, qRT-PCR has a good feasibility for detecting the expression level of hsa_circ_0087776 in serum.

### Hsa_circ_0087776 in serum of multiple myeloma was significantly reduced

**Figure 3.**
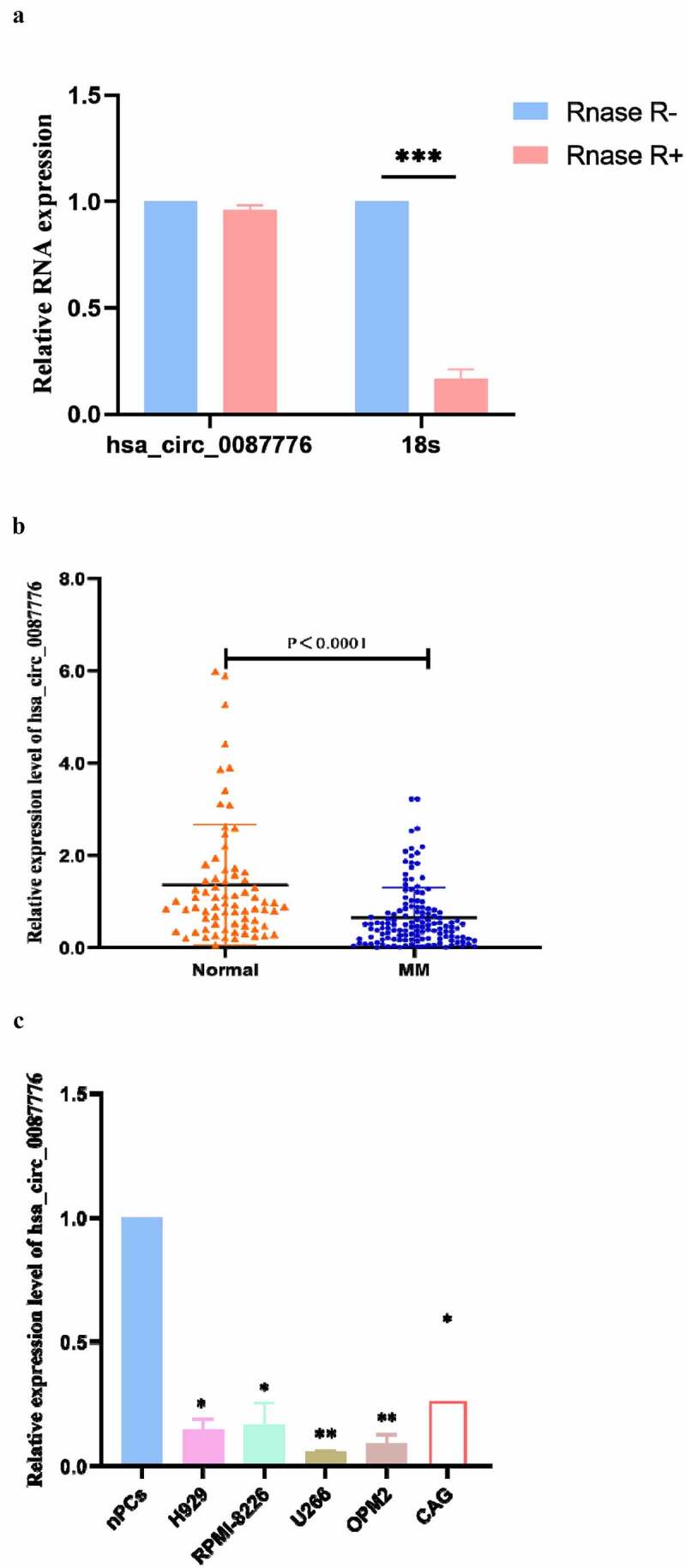
Detection of hsa _circ _0087776 relative expression in serum. (a) Hsa_circ-0087776 and 18s mRNA expression after treatment with RNase R. (b) Differential expression of hsa-circ-0087776 in serum of MM patients. (c) Differential expression of hsa-circ-0087776 in MM cell lines. (*P < 0.05; **P < 0.01; ***P < 0.001)

Firstly, we studied the annular stability, and found that hsa_circ_0087776 was highly resistant to RNase R and did not belong to a linear structure, shown in Figure 3(a). The expression level of hsa_circ_0087776 in serum of 136 MM patients and 74 healthy control patients was systematically analyzed, and the results showed that the expression level of hsa_circ_0087776 in serum of MM patients was significantly decreased (*P* < 0.0001) and there was significant difference, shown in Figure 3(b). The expression levels of hsa_circ_0087776 in MM cells (H929, U266, RPMI-8226, OPM2 and CAG) and human stromal cell (HS-5) were analyzed, and the results showed consistent expression in MM cells and serum with low expression. The expression difference was most obvious in U266, shown in Figure 3(c).

### Diagnostic efficacy of hsa_circ_0087776 in serum

**Figure 4.**
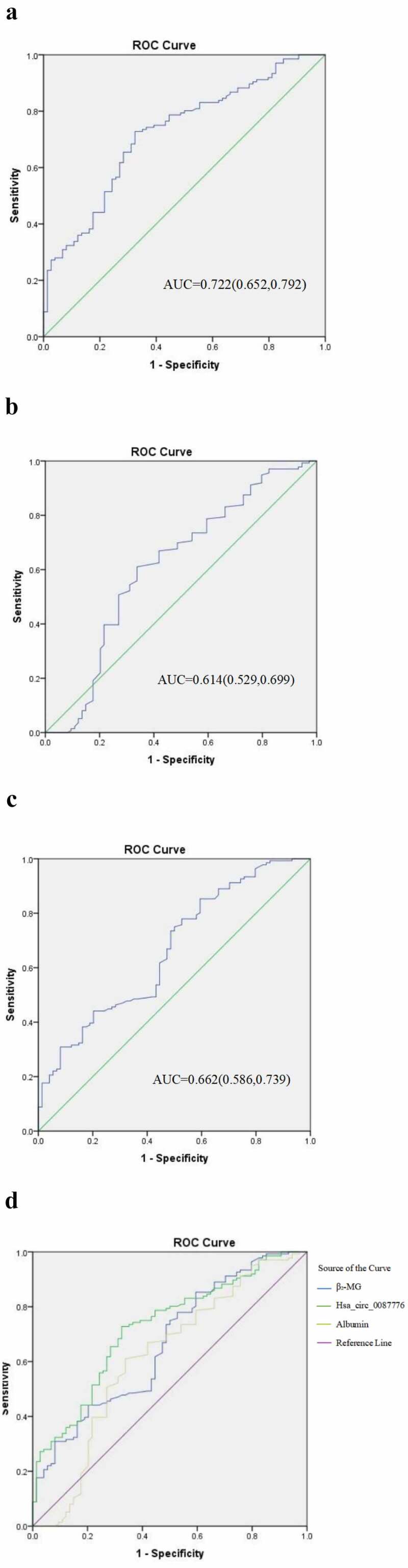
Evaluation of combined diagnostic efficacy of hsa _circ _0087776. (a) The ROC curve of serum hsa _circ _0087776. (b) The ROC curve of ALB. (c) The ROC curve of β2-MG. (d) By contrast, the diagnostic efficacy of serum hsa _circ _0087776 is remarkable

In order to better evaluate the diagnostic efficacy of hsa_circ_0087776 in clinical application, the relevant clinical data were collected and the ROC curve was analyzed to calculate the AUC area.The results showed that the AUC of hsa_circ_0087776 in serum was 0.722 (95%CI: 0.651, 0.792), shown in Figure 4(a), which was significantly higher than that of serum ALB (AUC = 0.614; 95%CI: 0.529, 0.699) and β₂-MG (AUC: 0.662; 95%CI: 0.586, 0.739), shown in (Figure 4(b) and (C), the above experiments proved the feasibility of using hsa_circ_0087776 as a new tumor marker with high diagnostic efficacy, shown in Figure 4(d).

### The expression of hsa_circ_0087776 changed before and after chemotherapy

**Figure 5.**
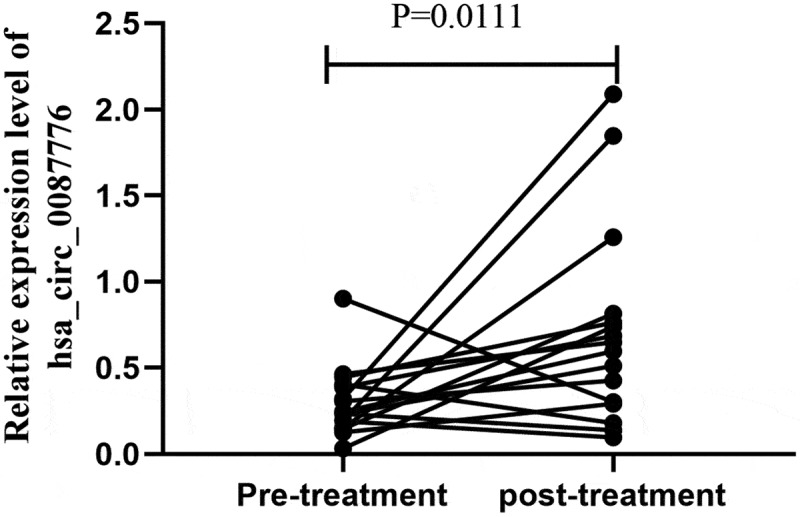
Changes in the expression of hsa_circ_0087776 before and after treatment (*P* = 0.0111)

Sixteen serum samples of MM patients before and after chemotherapy were collected, and the expression level of hsa_circ_0087776 after chemotherapy was significantly higher than that before chemotherapy (*P* < 0.001), shown in Figure 5, which provides a possibility for the detection of tumor dynamic changes and prognosis.

### Hsa_circ_0087776, albumin and beta-2-microglobulin combined diagnostic performance

According to statistical analysis, it was found that the diagnostic sensitivity of hsa_circ_0087776 was significantly higher than that of albumin (ALB) and Beta-2-microglobulin (β₂-MG), and the combined diagnosis of the three could significantly improve the diagnostic sensitivity of MM patients, shown in Table 2, which was helpful to improve the efficiency of diagnosis.

**Table 2.** Use of Hsa_circ_ 0087776, ALB, and β₂-MG levels to distinguish MM patients from healthy participants

	SEN (%)	SPE (%)	ACCU (%)	PPV (%)	NPV (%)
β₂-MG	32.4%	83.8%	50.5%	78.6%	40.3%
	(44/136)	(62/74)	(106/210)	(44/56)	(62/154)
Alb	33.1%	47.3%	38.1%	53.6%	27.8%
	(45/136)	(35/74)	(80/210)	(45/84)	(35/126)
Hsa_circ_ 0087776	72.8%	67.6%	71.0%	80.5%	57.5%
	(99/136)	(50/74)	(149/210)	(99/123)	(50/87)
β₂-MG + Hsa_circ_ 0087776	82.4%	56.8%	73.3%	77.8%	63.6%
	(112/136)	(42/74)	(154/210)	(112/144)	(42/66)
Alb + Hsa_circ_ 0087776	83.1%	35.1%	66.2%	70.0%	53.1%
	(113/136)	(26/74)	(139/210)	(113/162)	(26/49)
Alb + Hsa_circ_ 0087776 + β₂-MG	88.2%	31.1%	68.1%	70.2%	41.0%
	(120/136)	(23/74)	(143/210)	(120/171)	(16/39)

Note: Sensitivity (SEN); specificity (SPE); overall accuracy (ACCU); positive predictive value (PPV); negative predictive value (NPC)

### Correlation between hsa_circ_0087776 and clinical indicators

In this study, 136 patients with MM were divided into high expression group (n = 68) and low expression group (n = 68) according to the median. And according to the clinical indicators according to gender, age, M protein, light chain, HGB, total protein, albumin, globulin, β₂-MG and complications (renal damage and bone damage) for clinical grouping, statistical correlation.The results showed that hsa_circ_0087776 was significantly correlated with renal damage (*P* = 0.040) and *P* < 0.05, shown in Table 3. There was no significant difference (*P* > 0.05) with other indexes.

**Table 3.** Correlation between hsa_circ_ 0087776 expression and clinicopathologic features of MM patients

Clinical Characteristics	Case Number	hsa_circ_ 0087776		*Pearson χ^2^*	*P-value*
		Low Expression	High Expression		
Gender				2.944	0.860
Male	66	38	28		
Female	70	30	40		
Age				0.268	0.605
<65	75	39	36		
≥65	61	29	32		
M protein				1.121	0.290
IgA	84	39	45		
IgG	52	29	23		
Light chain				0.472	0.492
λ	64	30	34		
κ	72	38	34		
HGB (g/L)				0.138	0.710
<120	94	46	48		
≥120	42	22	20		
Total protein				0.287	0.592
Normal	97	42	45		
Abnormal	49	26	23		
Albumin				0.538	0.463
Normal	92	44	48		
Abnormal	44	24	20		
Globulin				0.282	0.595
Normal	85	41	44		
Abnormal	51	27	24		
β₂-MG (μg/L)				0.034	0.854
<3.5	93	46	47		
≥3.5	43	22	21		
Renal injury				4.225	0.040*
Yes	41	26	15		
No	95	42	53		
Bone injury				0.033	0.857
Yes	89	45	44		
No	47	23	24		

Note: Statistical analyses were carried out using Pearson χ^2^ test. *P < 0.05 was considered significant

### Independent correlation analysis of multiple myeloma patients

Univariate analysis was performed on the pathological parameters related to MM-ISS staging and prognosis, including β₂-MG, albumin, renal injury, bone injury and hsa_circ_0097776. The corresponding results was shown in Table 4. The results showed that β₂-MG (*P* = 0.002), albumin (*P* = 0.001), bone injury (*P* < 0.001), and hsa_circ_0097776 (*P* < 0.001) were significantly associated with MM. Multivariate analysis was performed on these related variables.The results showed that β₂-MG (*P* = 0.003), albumin (*P* < 0.001), bone injury (*P* < 0.001), hsa_circ_0097776 (*P* < 0.001) were independently associated with ISS stage and prognosis in MM patients.

**Table 4.** Univariate and multivariate analysis of MM-ISS staging and prognostic factors

Clinicopathological Parameters	Univariate analysis		Multivariate analysis	
	HR (95% CI)	*P*-value	HR (95% CI)	*P*-value
β₂-MG (<3.5 μg/ml vs ≥3.5 μg/ml)	2.471 (1.403, 4.351)	0.002**	2.711 (1.394, 5.271)	0.003**
Albumin (Outlier interval vs Rrference interval)	0.429 (0.265, 0.696)	0.001**	0.327 (0.183, 0.585)	< 0.001***
Renal injury (Yes vs No)	1.088 (0.865, 1.396)	0.477	-	-
Bone injury (Yes vs No)	5.482 (3.289, 9.136)	< 0.001***	5.224 (2.971, 9.187)	< 0.001***
hsa_circ_0087776 (low expresssion vs high expression)	4.228 (2.564, 6.974)	< 0.001***	4.191 (2.371, 7.406)	< 0.001***

Note: Univariate and multivariate analyses were performed by logistic regression analysis.

*P < 0.05, **P < 0.01, ***P < 0.001. CI:confidence interval.

## Discussion

In the current social environment, MM is characterized by genetic complexity and genetic heterogeneity, and the human leukocyte antigen (HLA) molecule was highly expressed [25]. With the emergence of serological markers and the advent of targeted drugs, the median survival time has increased from 2 years to 10 years [26]. However, the current diagnosis of MM still requires a comprehensive diagnosis method of immunotyping after bone marrow puncture, serological indicators and imaging, which is time-consuming and invasive, and is not suitable for physical examination and detection of disease changes in healthy patients, and it is difficult to realize the treatment principles of diagnosis, treatment and noninvasive detection. Serological tumor markers belong to noninvasive examination, which can effectively reduce the rate of misdiagnosis and missed diagnosis caused by tumor heterogeneity, and also realize real-time tumor monitoring to a certain extent.

In the current scientific research environment, the research on non-coding RNA is increasingly hot, and the research on the mechanism of non-coding RNA is more and more in-depth. Studies have shown that circRNA can play a role through sponge-mediated miRNA, through the construction of competitive endogenous RNA (ceRNA) network [27]. A large number of studies have shown that circ-RNA is involved in the development and prognosis of many kinds of tumors [28,29]. CircSEC24A provides a potential target for the clinical diagnosis and treatment of Osteoarthritis (OA) through miR-142-5p/SOX5 [30]. Circ-NEIL3 provides a novel marker for the diagnosis and prognosis of pancreatic ductal adenocarcinoma (PDAC) via miR-320-5p/AdAR1 [28]. Circ-HIPK3 provides a new approach for the diagnosis and treatment of oral squamous cell carcinoma through miR-381-3p/YAP1 [31]. It is proved that circRNA has a wide development prospect.

In this study, the differential expression of hsa_circ_0087776 in serum was first proposed.First of all, we chose 18s rRNA as the internal reference, and the qRT-PCR test results showed that hsa_circ_0087776 was single-peak specific, and the CV values of intra-batch and inter-batch differences were all less than 5%, which proved that hsa_circ_0087776 had good annular stability, accuracy, precision and stability. The above results demonstrate the feasibility of the experimental method. Subsequently, we detected the expression level of hsa_circ_0087776 by qRT-PCR, and found that the expression level of hsa_circ_0087776 was low in serum and cells, suggesting the possibility of its existence as a tumor suppressor gene in cells. In order to further verify its diagnostic efficacy as a clinical indicator, the area under ROC curve suggested that its diagnostic efficiency was better than that of ALB and β₂-MG, and the combined diagnosis of the three significantly improved the diagnostic sensitivity of MM. And statistical analysis of clinical indicators showed that there was a significant difference in the expression of renal injury compared with the adverse prognosis of MM. The prognosis of MM patients is affected by many factors, such as international staging system, cytogenetic abnormality, underlying disease and age [32], studies have shown that IFI16 can be used as a biomarker for poor prognosis in MM [33]. Later, we will further follow up and monitor the survival and prognosis of MM patients. The experiment found that hsa_circ_0087776 can affect the probability of renal injury in MM patients to a certain extent, thus affecting the prognosis of patients. After univariate and multivariate analysis, hsa_circ_0087776 expression level in MM patients was compared with clinicopathological parameters and disease stage, and it was found that hsa_circ_0087776 could be an independent predictor of MM. However, whether it can be used as a new serological diagnostic indicator and as an aid to the combined diagnosis of other indicators remains to be further studied.

In this experiment, in order to detect the diagnosis and treatment in the process of dynamic change of MM patients with serum collected before and after treatment, the results show that for most of most patients, their hsa_circ_0087776 expressions after chemotherapy are on the rise, while the hsa_circ_0087776 expressions of some patients decline. However, owing to the insufficient sample size, exclude the possibility of individual difference is not excluded. In future we will continue to research by influencing the expression mechanism, enlarging the sample size, and perfecting the relevant samples.

## Conclusion

In conclusion, hsa_circ_0087776 may act as a tumor suppressor gene in MM patients. Through qRT-PCR analysis of its was specific expression, that is, serum hsa_circ_0087776 may be used as a new biomarker for auxiliary diagnosis. Combined detection of serum β₂-MG, ALB level can further improve the detection rate of MM, reduce the rate of missed diagnosis. However, due to the small sample base and limited collection range of the disease, it is inevitable to be affected by the regional limitations and contingency of the disease. Therefore, it is necessary to increase the sample size to prove the above conclusions. In addition, the expression of MM in tissues has not been further confirmed. In the following experiments, we will focus on the expression of hsa_circ_0087776 in tissues and its influence on the pathogenesis of MM, the prognosis and survival time of MM patients were followed up and further study the influence of hsa_circ_0087776 on the prognosis of the disease. Improve the study of related influencing mechanism and signal pathway.

## Data Availability

The datasets used and/or analyzed during the current study are available from the corresponding author on reasonable request.

## Research highlights

- hsa_circ_0087776 expression was decreased in serum of MM patients and MM cell, and it was obviously correlated with renal injury.
- hsa_circ_0087776 is a better prediction tool than ALB or β₂-MG.
- The sensitivity of hsa_circ_0087776 was significantly improved by combining with ALB and β₂-MG.
